# Characterising cognitive heterogeneity in individuals at clinical high-risk for psychosis: a cluster analysis with clinical and functional outcome prediction

**DOI:** 10.1007/s00406-021-01315-2

**Published:** 2021-08-16

**Authors:** Kate Haining, Ruchika Gajwani, Joachim Gross, Andrew I. Gumley, Robin A. A. Ince, Stephen M. Lawrie, Frauke Schultze-Lutter, Matthias Schwannauer, Peter J. Uhlhaas

**Affiliations:** 1 Institute of Neuroscience and Psychology, University of Glasgow, Glasgow, UK; 2 Institute of Health and Wellbeing, University of Glasgow, Glasgow, UK; 3 Institute for Biomagnetism and Biosignalanalysis, University of Münster, Münster, Germany; 4 Department of Psychiatry, University of Edinburgh, Edinburgh, UK; 5 Department of Psychiatry and Psychotherapy, Medical Faculty, Heinrich Heine University, Düsseldorf, Germany; 6 Department of Psychology and Mental Health, Faculty of Psychology, Airlangga University, Surabaya, Indonesia; 7 University Hospital of Child and Adolescent Psychiatry and Psychotherapy, University of Bern, Bern, Switzerland; 8 Department of Clinical Psychology, University of Edinburgh, Edinburgh, UK; 9 Department of Child and Adolescent Psychiatry, Charité Universitätsmedizin, Berlin, Germany

**Keywords:** Clinical high-risk, First-episode psychosis, Cognition, Cluster analysis, Heterogeneity, Functional outcome

## Abstract

Schizophrenia is characterised by cognitive impairments that are already present during early stages, including in the clinical high-risk for psychosis (CHR-P) state and first-episode psychosis (FEP). Moreover, data suggest the presence of distinct cognitive subtypes during early-stage psychosis, with evidence for spared vs. impaired cognitive profiles that may be differentially associated with symptomatic and functional outcomes. Using cluster analysis, we sought to determine whether cognitive subgroups were associated with clinical and functional outcomes in CHR-P individuals. Data were available for 146 CHR-P participants of whom 122 completed a 6- and/or 12-month follow-up; 15 FEP participants; 47 participants not fulfilling CHR-P criteria (CHR-Ns); and 53 healthy controls (HCs). We performed hierarchical cluster analysis on principal components derived from neurocognitive and social cognitive measures. Within the CHR-P group, clusters were compared on clinical and functional variables and examined for associations with global functioning, persistent attenuated psychotic symptoms and transition to psychosis. Two discrete cognitive subgroups emerged across all participants: 45.9% of CHR-P individuals were cognitively impaired compared to 93.3% of FEP, 29.8% of CHR-N and 30.2% of HC participants. Cognitively impaired CHR-P participants also had significantly poorer functioning at baseline and follow-up than their cognitively spared counterparts. Specifically, cluster membership predicted functional but not clinical outcome. Our findings support the existence of distinct cognitive subgroups in CHR-P individuals that are associated with functional outcomes, with implications for early intervention and the understanding of underlying developmental processes.

**Supplementary Information:**

The online version contains supplementary material available at 10.1007/s00406-021-01315-2.

## Introduction

Schizophrenia is a debilitating psychiatric disorder characterised by psychotic symptoms, including hallucinations and delusions, as well as impairments in cognition, sensory processing and psychosocial functioning [1, 2]. Cognitive impairments span several domains including processing speed, working memory, executive functions, attention and social cognition [3, 4]. Schizophrenia is preceded, in the majority of cases, by a clinical high-risk for psychosis (CHR-P) state lasting approximately 5–6 years [5]. CHR-P status is determined using ultra-high-risk (UHR) criteria, encompassing attenuated psychotic symptoms (APS), brief frank psychosis and functional decline with genetic risk [6], as well as basic symptom criteria that involve self-experienced perceptual and cognitive disturbances [7, 8]. CHR-P individuals are also characterised by widespread cognitive impairments intermediate between healthy controls (HC) and first-episode psychosis (FEP) patients [9, 10]. These impairments, especially in attention, working memory and declarative memory, are more pronounced in CHR-P individuals who later transition to psychosis [11]. However, cognitive performance within the CHR-P state is highly variable with small-to-large effect size impairments (Cohen’s *d* = − 0.35 to − 0.84) in those who transition to psychosis and small-to-medium impairments (*d* = − 0.26 to − 0.67) in those who do not [9]. Accordingly, novel approaches may be required to identify subtypes of CHR-P participants with different cognitive profiles, with possible implications for the understanding of underlying pathophysiology and accurate prediction of outcomes.

Data-driven approaches, such as cluster analysis, classify individuals according to levels and patterns of performance, rather than pre-determined grouping criteria [12]. Cognitive subgroups have successfully been identified in cross-diagnostic samples, comprising individuals with schizophrenia-spectrum disorders or mood disorders [12–16]. These findings support the existence of a range of cognitive impairments across different syndromes with evidence for two [14], three [13, 16] and four [12, 15] cognitive subgroups.

Furthermore, emerging evidence suggests that cluster analysis can identify phenotypes that relate more closely to specific clinical and functional trajectories than existing diagnostic categories [17]. Indeed, such approaches have highlighted poorer functioning and greater symptom severity in cognitively impaired vs. cognitively spared subgroups in schizophrenia-spectrum populations [12, 15, 18–21]. Moreover, subgroups with impaired cognition have also been associated with reductions in brain volume [22, 23] and different profiles of treatment response [24].

There is preliminary evidence for similar profiles of cognitive impairment in FEP patients, with little consensus on the number of emergent clusters [25–28]. Wenzel et al. [28] and Reser et al. [25] identified two and four cognitive subgroups in FEP patients, respectively, with high negative symptom severity and low premorbid IQ characteristic of the most cognitively impaired subgroup. Interestingly, Uren et al. [27] and Sauvé et al. [26] both obtained a three-cluster solution and found that 28% and 54% of FEP participants, respectively, aggregated with HCs in the cognitively spared subgroup, supporting the existence of an FEP subgroup with intact cognitive functioning. According to Uren et al. [27], cluster membership was associated with symptom severity and functioning from baseline to 6 months, highlighting the potential utility of cognitive clustering for prognosis and early intervention.

To our knowledge, only one study has used cluster analysis to examine cognitive profiles in CHR-P participants. Velthorst et al. [29] derived four distinct cognitive subgroups, whereby 44% of CHR-P participants were significantly or mildly impaired and 56% displayed average or above average cognitive scores. In addition, cognitive subgroups yielded prognostic information with cluster membership predicting conversion to psychosis over a 30-month follow-up period. However, this study did not examine the predictive utility of cognitive subgroups in relation to global functioning or symptom persistence and did not include any measures of social cognition. Furthermore, it is unclear whether these findings from help-seeking CHR-P participants would generalise to more representative samples recruited outside clinical pathways.

To address these important questions, we sought to identify cognitive clusters in a sample of CHR-P and FEP participants, primarily recruited from the community, alongside individuals who did not fulfil CHR-P criteria but were characterised by affective and substance use disorders (CHR-Ns) and HCs. Specifically, we performed cluster analysis on principal components derived from both neurocognitive and social cognitive measures. We then examined the distribution of diagnostic groups across clusters and investigated whether cognitive subgroups were associated with clinical and functional variables at baseline and follow-up in the CHR-P group. Given previous findings in CHR-P and FEP samples [25–27, 29], we hypothesised the existence of at least three distinct cognitive profiles. In addition, we expected CHR-P individuals with pronounced cognitive deficits to exhibit the poorest functioning and greatest symptom severity at baseline and follow-up as well as cluster membership to predict clinical and functional outcomes in the CHR-P group.

## Methods

### Participants

Participants were recruited through the ongoing Youth Mental Health Risk and Resilience (YouR) study [30] which seeks to identify neurobiological and psychological mechanisms and predictors of psychosis risk. CHR-P participants from the general population were recruited through an online-screening approach (www.your-study.org.uk) [31]. FEP and CHR-N participants were also recruited using this method while HCs were obtained from a volunteer database. A smaller number of CHR-P and FEP individuals were also recruited via referrals from clinical services in NHS Greater Glasgow and Clyde and NHS Lothian as well as student counselling services. Ethical approval was obtained from the West of Scotland Research Ethics Service and the University of Glasgow. All participants provided written informed consent.

Baseline data were available for 146 CHR-P participants, 15 participants with first-episode psychosis (FEP), 47 participants who did not fulfil CHR-P criteria (CHR-Ns) and 53 healthy controls (HCs). Unlike HCs, CHR-N participants met criteria for mood and anxiety disorders as well as substance use. Thus, the inclusion of the CHR-N group allowed us to potentially disentangle the impact of psychiatric comorbidity from the CHR-P state since mood and anxiety disorders are common in this population [32]. Referred participants comprised 11.0% of the CHR-P sample and 46.7% of the FEP sample. One hundred and twenty-two CHR-P participants (83.6%) also completed a follow-up session 6- and/or 12-months later.

Previous publications by our group have reported baseline demographic, clinical, functional and cognitive data from similar or smaller samples [31, 33–35]*.*

### Baseline assessments

To establish CHR-P criteria, participants received the positive scale of the Comprehensive Assessment of At-Risk Mental States (CAARMS) [6] and the Cognitive Disturbances (COGDIS) and Cognitive-Perceptive Basic Symptoms (COPER) items of the Schizophrenia Proneness Instrument, Adult version (SPI-A) [36].

Participants were recruited into the CHR-P group if they met one or both SPI-A criteria (i.e. COGDIS, COPER) and/or at least one of the following CAARMS criteria: APS, genetic risk and functional deterioration (GRFD), brief limited intermittent psychotic symptoms (BLIPS). FEP criteria were established using the Structured Clinical Interview for DSM-IV (SCID) [37] and the Positive and Negative Syndrome Scale (PANSS) [38].

Cognitive assessments consisted of the Brief Assessment of Cognition in Schizophrenia (BACS) [39] and three tasks from the Penn Computerized Neurocognitive Battery (CNB) [40]: the Continuous Performance Test, the N-Back Test and the Emotion Recognition Task which provide measures of accuracy and response time (RT) for attention, working memory and emotion recognition respectively (Supplementary Table 1). Furthermore, with the exception of the FEP group, all participants were assessed with the Mini-International Neuropsychiatric Interview (MINI) [41], Global Assessment of Functioning (GAF) scale from the DSM-IV-TR, Global Functioning: Social (GF: Social) and Role (GF: Role) scales [42], Premorbid Adjustment Scale (PAS) [43] and National Adult Reading Test (NART) [44].

### Clinical and functional outcome

CHR-P participants were invited for follow-up interviews at 6- and 12-months. These involved the positive scale of the CAARMS as well as the GAF, GF: Social and GF: Role scales. Based on the most recent GAF score, CHR-P participants were divided into good functional outcome (GAF ≥ 65) and poor functional outcome (GAF < 65) groups, in line with previous research [45, 46]. CAARMS persistence was operationalised as meeting APS criteria at both baseline and the latest follow-up assessment. Transitions to psychosis, recorded over a 36-month follow-up period, were also defined according to CAARMS criteria and subsequently followed up with the Structured Clinical Interview for DSM-IV (SCID) [37] to establish the specific psychosis diagnoses.

### Statistical analysis

Data were analysed using R version 4.0.1 [47] with statistical significance set at *p* < 0.05 (two-tailed). Overall, 0.48% of the data (52 of 10,904 values) were missing and imputed by Bayesian imputation.

In line with Keefe et al. [48], BACS raw scores for each cognitive domain were converted into standardized z-scores using the means and standard deviations (SDs) of sex-specific HCs. For consistency, CNB raw accuracy and RT scores were calculated in the same way, albeit without correction for sex. RT z-scores were multiplied by − 1, to produce speed values where, as for accuracy, higher scores reflect better performance. CNB efficiency scores were then generated for each domain by taking the arithmetic mean of the accuracy and RT z-scores. Outliers beyond ± 5.0 z-scores were curtailed to values of + 5.0 or − 5.0. NART-derived estimates of premorbid full-scale IQ were obtained using a recently re-standardised calculation [49]. CAARMS severity was calculated by multiplying the global score by the frequency score for each domain and summing these products [50] while SPI-A severity was calculated by summing the frequency scores for each basic symptom.

In the first step, a principal component analysis (PCA) was conducted on 20 cognitive tests with oblique (oblimin) rotation, so as to allow for possible correlations between the factors, using the *psych* [51] and *GPArotation* [52] packages. Data suitability for PCA was assessed with the Kaiser–Meyer–Olkin (KMO) measure of sampling adequacy [53, 54] and Bartlett's test of sphericity [55]. To determine the appropriate number of principle components to extract, we used the Kaiser criterion of eigenvalues > 1 [56] as well as scree plot inspection [57]. Cronbach’s α was used to determine the internal consistency of data.

In the second step, we evaluated the clustering tendency of our data as well as the optimal clustering approach. Clustering tendency of the resulting component scores was assessed using the Hopkins (H) statistic via the *clustertend package* [58]. A value close to 1 indicates uniformly distributed data while highly clustered data yields a value close to 0. To identify the optimal clustering algorithm and number of clusters, we used the *clValid* package [59] which simultaneously compares the different clustering solutions in terms of validation measures. We tested for the presence of two to six clusters, implementing three clustering methods: (1) k-means, (2) partitioning around medoids (PAM) and (3) agglomerative hierarchical clustering. Internal validation measures were calculated as connectivity, silhouette width and Dunn index. Stability validation measures comprised the average proportion of non-overlap (APN), the average distance (AD), the average distance between means (ADM) and the figure of merit (FOM). Whereas internal validation measures evaluate the connectedness, compactness and separation of the different clusters, stability validation measures assess the consistency of a clustering result by comparing it with the clusters obtained after removing each column, one at a time. In general, smaller values reflect better performance, with the exception of silhouette width and Dunn index where larger values are preferable. This information was used to inform the third step whereby data-driven agglomerative hierarchical clustering was applied to the component scores via the *stats* package [47], using Ward’s method and squared Euclidean distance, to produce two clusters. Cross-validated linear discriminant analysis, using the 20 original standardised cognitive scores as independent variables, was performed with the *caret* package [60] to evaluate the classification accuracy of the final clustering solution.

For the CHR-P group, the resulting clusters were compared on demographic, functional, clinical and cognitive characteristics using Welch’s *t* tests, Mann–Whitney *U* tests, Pearson’s chi‐squared tests and Fisher's exact tests. We also conducted a series of hierarchical multiple linear regression analyses to examine effects of cluster membership on cognitive domains and functional variables after controlling for the potential effects of clinical (CAARMS and SPI-A severity) and demographic (age, sex, education) variables to examine the possibility that differences by cluster were better accounted for by overall symptom severity or demographic characteristics. In these models, clinical and demographic variables were entered in step 1 and cluster membership was entered in step 2. Binary logistic regression analyses were also employed to determine whether cluster membership could predict clinical and functional outcomes. The overall variance explained was measured by the Nagelkerke pseudo R^2^ statistic (R^2^N) while diagnostic accuracy was determined using the area under the receiver operating characteristic (ROC) curve (AUC).

## Results

### Demographic data

CHR-P individuals had significantly fewer years of education, greater symptom severity, higher likelihood of comorbid mood and anxiety disorders and poorer functioning compared to CHR-N and HC participants (Table 1). Relative to the total sample, CHR-P individuals were also significantly younger while FEP patients displayed significantly higher CAARMS severity, antipsychotic and anxiolytic medication use as well as poorer global functioning. Among the CHR-P group, 43 (29.5%) met CAARMS criteria, 34 (23.3%) met SPI-A criteria and 69 (47.3%) met both. Moreover, the FEP group comprised participants with SCID DSM-IV schizophrenia (*n* = 10; 66.7%), psychotic disorder not otherwise specified (*n* = 3; 20.0%), schizoaffective disorder (*n* = 1; 6.7%) and schizophreniform disorder (*n* = 1; 6.7%).

**Table 1:** Demographic, clinical and functional characteristics of the total sample (*N* = 261) at baseline

	CHR-P (1) (*N* = 146)	FEP (2) (*N* = 15)	CHR-N (3) (*N* = 47)	HC (4) (*N* = 53)	*p*	Effect size^a^	Post hoc test^b^
Age (years), mean (SD)	21.47 (4.22)	24.40 (4.37)	22.94 (4.80)	22.42 (3.36)	0.003	η^2^_*p*_ = 0.051	2,3,4 > 1
Sex, female *n* (%)	104 (71.2)	7 (46.7)	30 (63.8)	36 (67.9)	0.241	*V* = 0.127	
Education (years), mean (SD)	15.12 (3.10)	15.25 (2.84)	16.45 (3.44)	16.47 (2.85)	0.010	η^2^_*p*_ = 0.043	3,4 > 1
CAARMS severity, median (range)	28 (0–74)	79 (38–122)	6 (0–24)	0 (0–12)	< 0.001	η^2^_*p*_ = 0.404	2 > 1 > 3 > 4
SPI-A severity, median (range)	7 (0–74)	15 (0–109)	0 (0–7)	0 (0–2)	< 0.001	η^2^_*p*_ = 0.336	1,2 > 3,4
GAF, median (range)	58 (21–95)	41 (18–79)	70 (43–94)	88 (67–97)	< 0.001	η^2^_*p*_ = 0.343	4 > 3 > 1 > 2
Social functioning (current), median (range)	8 (3–10)		8 (6–9)	9 (8–10)	< 0.001	η^2^_*p*_ = 0.229	4 > 3 > 1
Role functioning (current), median (range)	8 (3–9)		8 (5–9)	9 (5–9)	< 0.001	η^2^_*p*_ = 0.199	4 > 3 > 1
PAS average, median (range)	1.28 (0–3.43)		0.86 (0–3.86)	0.43 (0–1.64)	< 0.001	η^2^_*p*_ = 0.189	1 > 3 > 4
Comorbidity, *n* (%)							
Anxiety disorder	104 (71.2)		22 (46.8)	0 (0)	< 0.001	*V* = 0.568	1 > 3 > 4
Mood disorder	97 (66.4)		14 (29.8)	0 (0)	< 0.001	*V* = 0.552	1 > 3 > 4
Alcohol abuse/dependence	46 (31.5)		11 (23.4)	0 (0)	< 0.001	*V* = 0.297	1,3 > 4
Substance abuse/dependence	24 (16.4)		3 (6.4)	0 (0)	0.002	*V* = 0.221	1 > 4
Eating disorder	13 (8.9)		1 (2.1)	0 (0)	0.023	*V* = 0.170	1 > 4
Psychological treatment, *n* (%)							
Current	25 (17.1)	5 (33.3)	5 (10.6)	0 (0)	0.002	*V* = 0.243	2 > 3,4 and 1 > 4
Past	66 (45.2)	5 (33.3)	15 (31.9)	3 (5.7)	< 0.001	*V* = 0.323	1,2,3 > 4
Medication, *n* (%)							
Antidepressants	53 (36.3)	9 (60.0)	13 (27.7)	0 (0)	< 0.001	*V* = 0.354	2 > 3,4 and 1 > 4
Mood stabilisers	4 (2.7)	0 (0)	0 (0)	0 (0)	0.592	*V* = 0.111	
Antipsychotics	4 (2.7)	7 (46.7)	0 (0)	0 (0)	< 0.001	*V* = 0.526	2 > 1,3,4
Anxiolytics	10 (6.8)	5 (33.3)	1 (2.1)	0 (0)	< 0.001	*V* = 0.304	2 > 1,3,4

*CHR-P* clinical high-risk for psychosis, *FEP* first-episode psychosis, *CHR-N* clinical high-risk-negative, *HC* healthy control, *CAARMS* Comprehensive Assessment of At-Risk Mental States, *SPI-A* Schizophrenia Proneness Instrument, Adult version, *GAF* Global Assessment of Functioning, *PAS* Premorbid Adjustment Scale

^a^Effect sizes were eta squared (η^2^_*p*_) for Kruskal–Wallis *H* tests (small effect = 0.01, medium effect = 0.06, large effect = 0.14) and Cramer's V for Pearson’s chi-squared or Fisher–Freeman–Halton tests (small effect = 0.1, medium effect = 0.3, large effect = 0.5)

^b^1 = CHR-P, 2 = FEP, 3 = CHR-N, 4 = HC

### Principal component analysis

**Fig. 1:**
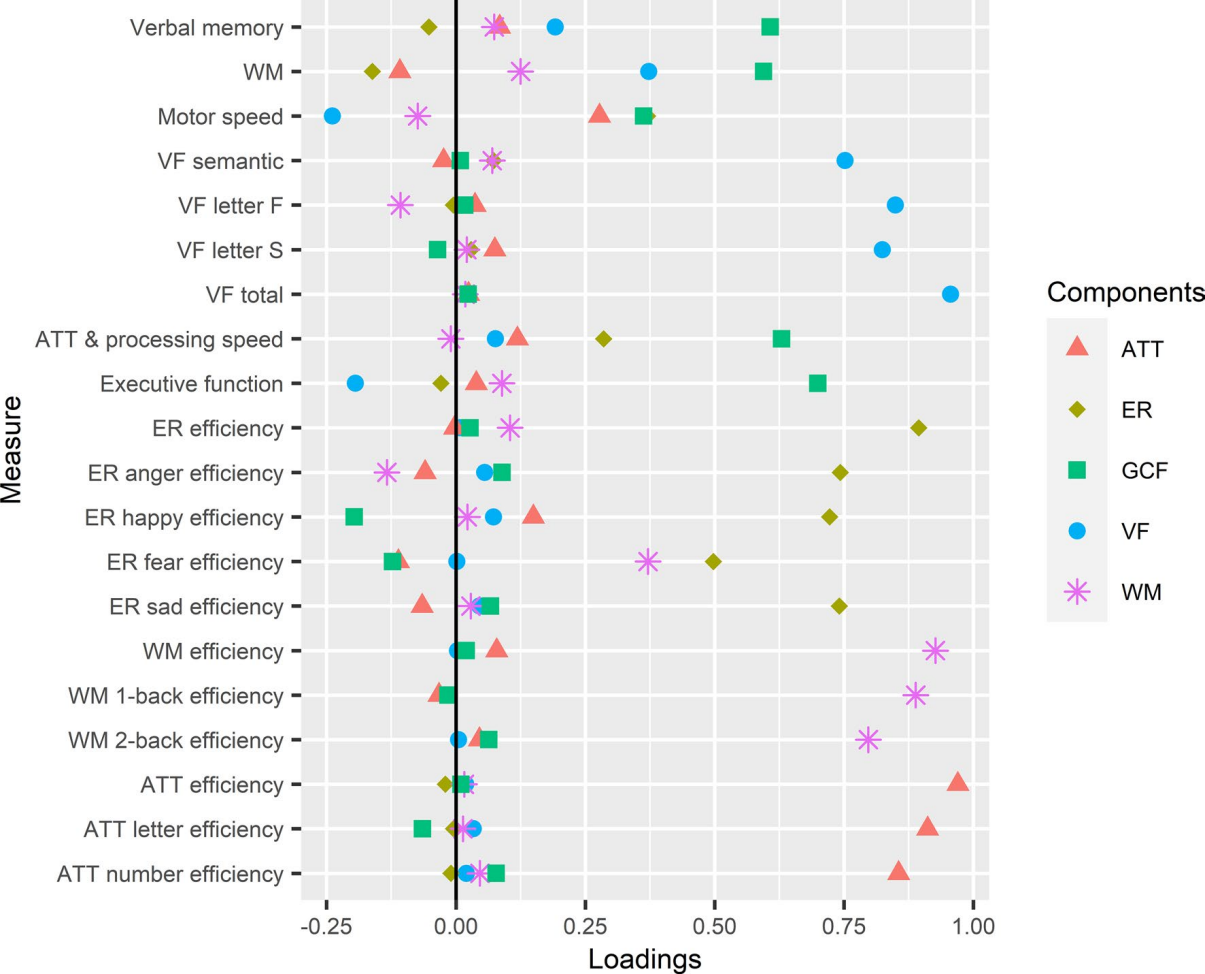
Component loading plot for the total sample (*N* = 261). *ATT* attention, *ER* emotion recognition, *GCF* general cognitive function, *VF* verbal fluency, *WM* working memory

The KMO measure verified the sampling adequacy for the PCA (KMO = 0.70) with all values for individual items ≥ 0.52, which is above the acceptable limit of 0.50. Bartlett’s test of sphericity, χ^2^ (190) = 3795.385, *p* < 0.001, indicated that correlations between items were sufficiently large for PCA. Five principal components were extracted and, in combination, explained 68% of the variance in cognitive performance (Fig. 1; Supplementary Tables 2 and 3). These were labelled verbal fluency (*α* = 0.89), emotion recognition (*α* = 0.82), attention (*α* = 0.93), working memory (*α* = 0.88) and general cognitive function (*α* = 0.68).

### Agglomerative hierarchical cluster analysis

The resulting dataset contained statistically meaningful clusters (*H* = 0.24). All internal validation measures and two out of four stability validation criteria favoured agglomerative hierarchical clustering with two clusters. The dendrogram was cut to produce two clusters and subjects were assigned cluster membership accordingly (Supplementary Fig. 1). Cluster 1 comprised 111 (42.5%) cognitively impaired participants while cluster 2 comprised 150 (57.5%) cognitively spared participants. Linear discriminant analysis with tenfold repeated (100 times) cross-validation, using the 20 original standardised cognitive scores as independent variables, confirmed that we were able to predict the cluster membership of new cases with a mean accuracy of 88.8%.

**Fig. 2:**
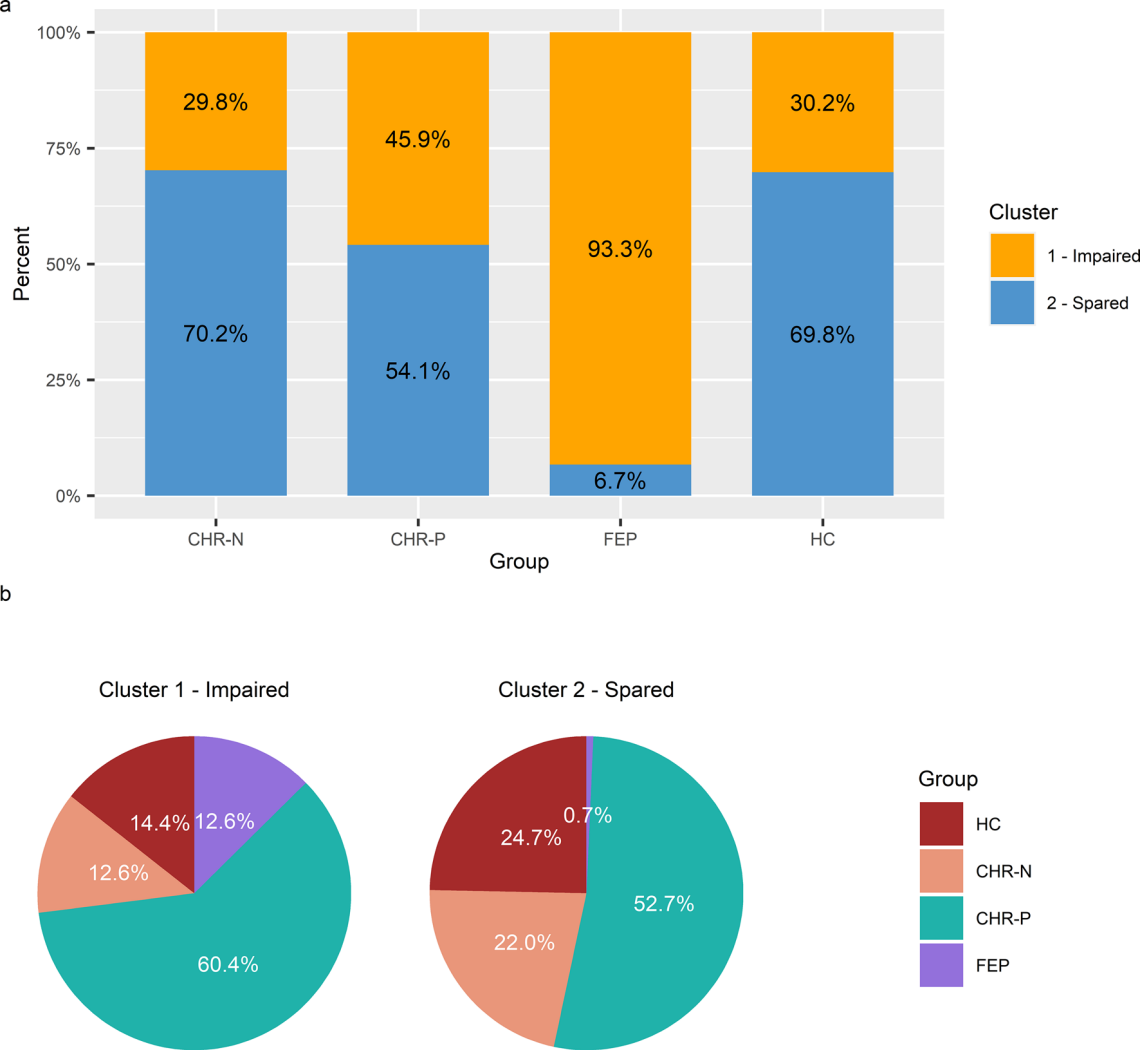
The distribution of **a** clusters within each diagnostic group and **b** diagnostic groups within each cluster for the total sample (*N* = 261). *CHR-P* clinical high-risk for psychosis, *FEP* first-episode psychosis, *CHR-N* clinical high-risk-negative; *HC* healthy control

Cluster 1 comprised 93.3% (*n* = 14) of FEP individuals and 45.9% (*n* = 67) of CHR-P participants (Fig. 2). In addition, similar percentages of CHR-N and HC individuals were assigned to cluster 1 (CHR-N: 29.8%; HC: 30.2%).

### Cluster comparisons at baseline

**Fig. 3:**
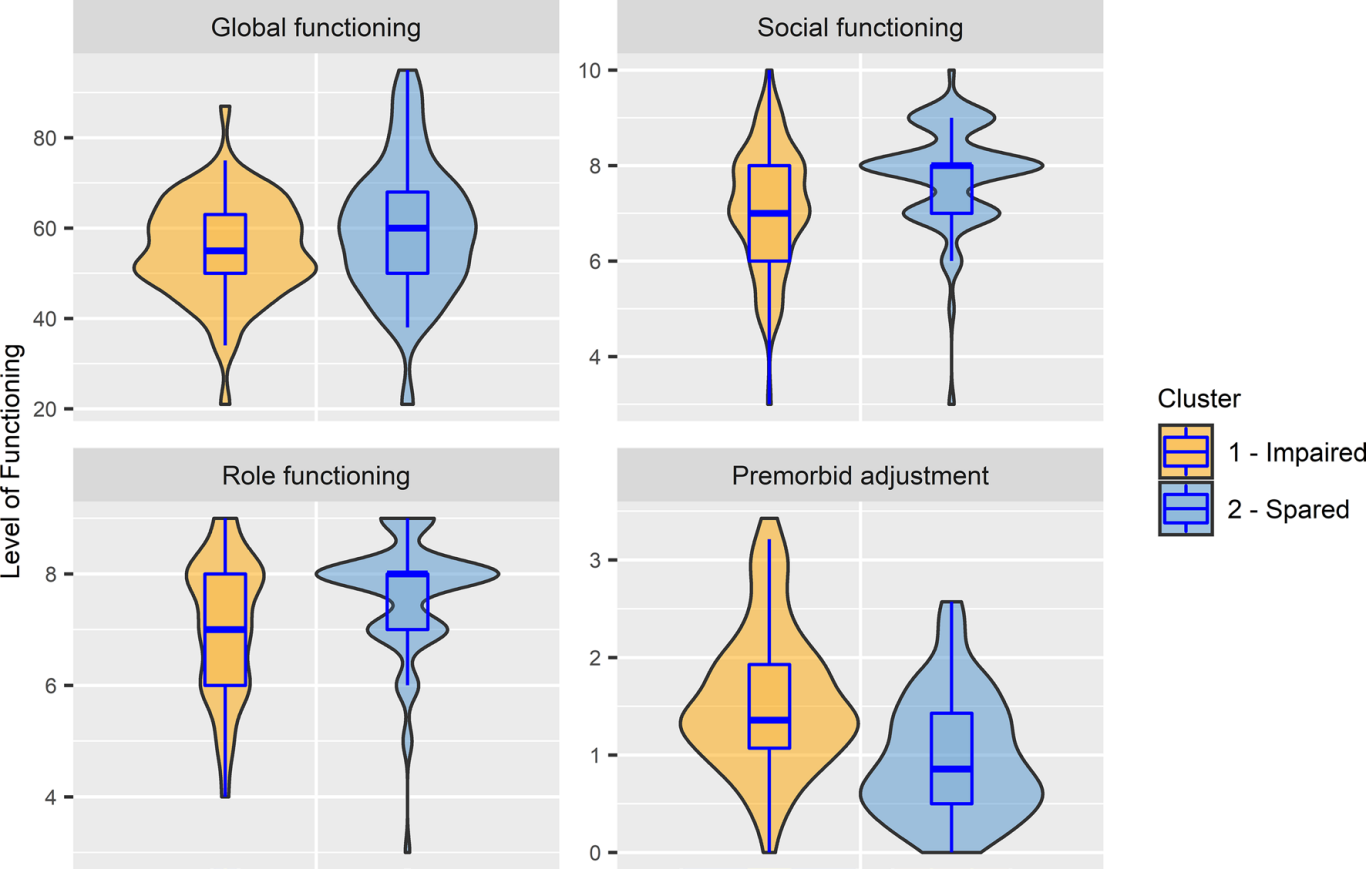
Level of functioning across cognitive clusters for the CHR-P group (*N* = 146)

CHR-P individuals in cluster 1 displayed significantly lower premorbid IQ and poorer performance across all 20 cognitive tests compared to those in cluster 2 (*p* < 0.01), with medium to large effect sizes (Supplementary Table 4), and were characterised by poorer social, role and premorbid functioning (*p* < 0.01) but not global functioning (Table 2; Fig. 3). Male CHR-P participants were also significantly more likely (*p* < 0.001*)* to be allocated to cluster 1 (47.8%) than cluster 2 (12.7%). After controlling for clinical symptoms and demographic characteristics, cluster membership remained significantly associated with premorbid IQ (*t* = 2.565; *p* = 0.011), all 20 cognitive domains (*t* = 2.033 to 7.166; *p* < 0.05), social functioning (*t* = 2.375; *p* = 0.019) and premorbid functioning (*t* = − 3.997; *p* < 0.001), but not role functioning (*t* = 1.548; *p* = 0.124). Furthermore, the proportion of CHR-P participants meeting CAARMS criteria, SPI-A criteria or both did not differ between the clusters (*p* = 0.667).

**Table 2:** Demographic, clinical and functional characteristics of the CHR-P group by cognitive cluster at baseline (*N* = 146) and follow-up (*N* = 122)

Baseline	Cluster 1	Cluster 2	*p*	Effect size^a^
	Impaired (*N* = 67)	Spared (*N* = 79)		
Age (years), mean (SD)	21.36 (4.63)	21.56 (3.86)	0.288	*r* = 0.088
Sex, female *n* (%)	35 (52.2)	69 (87.3)	< 0.001	ϕ = 0.386
Education (years), mean (SD)	14.96 (3.43)	15.25 (2.80)	0.421	*r* = 0.067
CAARMS severity, median (range)	29 (0–74)	28 (0–72)	0.212	*r* = 0.103
SPI-A severity, median (range)	6 (0–61)	7 (0–74)	0.883	*r* = 0.012
GAF, median (range)	55 (21–87)	60 (21–95)	0.094	*r* = 0.139
Social functioning (current), median (range)	7 (3–10)	8 (3–10)	< 0.001	*r* = 0.296
Role functioning (current), median (range)	7 (4–9)	8 (3–9)	0.002	*r* = 0.255
PAS average, median (range)	1.36 (0–3.43)	0.86 (0–2.57)	< 0.001	*r* = 0.405
Comorbidity, *n* (%)				
Anxiety disorder	49 (73.1)	55 (69.6)	0.640	ϕ = 0.039
Mood disorder	50 (74.6)	47 (59.5)	0.054	ϕ = 0.160
Alcohol abuse/dependence	18 (26.9)	28 (35.4)	0.266	ϕ = 0.092
Substance abuse/dependence	11 (16.4)	13 (16.5)	0.995	ϕ = 0.001
Eating disorder	4 (6.0)	9 (11.4)	0.252	ϕ = 0.095
Psychological treatment, *n* (%)				
Current	15 (22.4)	10 (12.7)	0.120	ϕ = 0.129
Past	27 (40.3)	39 (49.4)	0.273	ϕ = 0.091
Medication, *n* (%)				
Antidepressants	25 (37.3)	28 (35.4)	0.815	ϕ = 0.019
Mood stabilisers	2 (3.0)	2 (2.5)	10.000	ϕ = 0.014
Antipsychotics	3 (4.5)	1 (1.3)	0.333	ϕ = 0.098
Anxiolytics	4 (6.0)	6 (7.6)	0.754	ϕ = 0.032

Follow-Up	Cluster 1	Cluster 2	*p*	Effect size^a^
	Impaired (*N* = 57)	Spared (*N* = 65)		
GAF, median (range)	52 (21–88)	68 (33–88)	0.012	*r* = 0.227
Poor functional outcome, *n* (%)	41 (71.9)	31 (47.7)	0.007	ϕ = 0.246
Social functioning (current), median (range)	8 (2–10)	8 (4–9)	0.021	*r* = 0.209
Role functioning (current), median (range)	8 (4–9)	8 (5–9)	0.139	*r* = 0.134
CAARMS severity, median (range)	15 (0–71)	12 (0–82)	0.886	*r* = 0.013
CAARMS persistence, *n* (%)	17 (29.8)	21 (32.3)	0.768	ϕ = 0.027
Transitions^b^, *n* (%)	9 (15.8)	5 (7.7)	0.162	ϕ = 0.127

*CHR-P* clinical high-risk for psychosis, *CAARMS* Comprehensive Assessment of At-Risk Mental State, *SPI-A* Schizophrenia Proneness Instrument, Adult version, *GAF* Global Assessment of Functioning, *PAS* premorbid adjustment scale

^a^Effect sizes were Rosenthal's r for Mann–Whitney *U* tests and Phi (ϕ) for Pearson’s chi-squared or Fisher′s exact tests (small effect = 0.1, medium effect = 0.3, large effect = 0.5)

^b^19 non-transitioned CHR-P individuals have yet to reach the 3-year follow-up

### Cluster comparisons and outcome prediction at follow-up

CHR-P individuals in cluster 1 displayed significantly poorer global and social functioning at follow-up 6- and/or 12-months later compared to those in cluster 2 (*p* < 0.05). Within the CHR-P group, poor functional outcome was also significantly more likely (*p* = 0.007) in cluster 1 (71.9%) compared to cluster 2 (47.7%).

In a binary logistic regression analysis, cluster membership explained 8.0% of the variance in functional outcome (*p* = 0.007, AUC = 0.625, sensitivity = 56.9% and specificity = 68.0%). Based on the odds ratio, poor functional outcome was 2.81 times higher if participants were assigned to cluster 1 rather than cluster 2. This association remained significant after adjusting for GAF score at baseline (adjusted odds ratio = 2.52, *p* = 0.030). In contrast, cluster membership could not predict clinical outcomes in terms of CAARMS persistence (*p* = 0.768) or transition to psychosis (*p* = 0.170).

### Additional analyses

The PCA and cluster analysis were repeated following the exclusion of the small sample of FEP participants to verify the stability and interpretability of our results. Overall, results remained unchanged, albeit with slightly smaller effect sizes (see Supplementary Results and Supplementary Figs. 2–5).

## Discussion

Using a data-driven hierarchical clustering approach in conjunction with PCA, we identified a two-cluster solution, comprising a cognitively spared and cognitively impaired subgroup, in a sample consisting of CHR-P and FEP participants as well as CHR-N participants and HCs. While the majority of FEP individuals were assigned to the cognitively impaired cluster, CHR-P individuals were almost equally distributed. At both baseline and follow-up, CHR-P individuals classified as cognitively impaired displayed significantly poorer functioning than their cognitively spared counterparts with cluster membership able to predict functional but not clinical outcome.

### Hierarchical clustering on principal components

In the present study, PCA was applied prior to clustering to reduce data dimensionality, thereby reducing information redundancy and maximising explanatory variance [61]. Verbal fluency, emotion recognition, attention, working memory and general cognitive function were the five principal components that explained 68% of the variance in cognitive performance across the entire sample. Interestingly, Lam et al. [62] observed a similar cognitive component structure in both CHR-P and HC samples, indicating that our components constitute reproducible dimensions of cognitive performance.

The emergence of a two-cluster solution is in agreement with previous studies involving schizophrenia-spectrum disorders [14, 20, 28, 63, 64]. However, three- or four-cluster solutions are more typically reported in mixed samples of FEP and HC participants [26, 27]. Furthermore, the only study to investigate cognitive subgroups in CHR-P participants obtained a four-cluster solution [29]. It is possible that our two-cluster solution partially reflects the novel combination of FEP and CHR-P participants as well as the application of basic symptom criteria to recruit CHR-P individuals. Nevertheless, this solution has resulted from replicable cognitive components [62], supporting the validity of our findings.

Finally, it is important to note that the majority of CHR-P participants in the current study were recruited from the community and not through dedicated clinical pathways. Community-recruitment strategies represent an important aspect of early detection and intervention [65, 66]. Indeed, there may be a substantial number of young people at CHR-P in the community who are not seen by specialised early detection services [65]. Therefore, community-recruitment strategies are particularly advantageous in their ability to detect more representative samples, ensuring that findings can be generalised to the entire population of individuals at CHR-P.

### Characterising within-group cognitive heterogeneity

In line with Velthorst et al. [29], our CHR-P group exhibited substantial cognitive heterogeneity, with 45.9% of individuals assigned to the cognitively impaired subgroup. On the other hand, cognitive heterogeneity was less apparent in our FEP group, contrasting with previous findings in larger samples [26, 27]. Approximately 16% fewer CHR-N participants were classified as cognitively impaired relative to CHR-P participants, indicating that cognitive impairment is somewhat more prevalent in the CHR-P state. Interestingly, a considerable proportion of HCs (30.2%) were also allocated to the cognitively impaired subgroup, supporting previous findings [26]. Overall, these results support the notion of a cognitive continuum [12, 16, 67], at least among CHR-P, CHR-N and HC populations.

### Cluster comparisons in the CHR-P group

Cognitively impaired CHR-P individuals displayed significantly poorer performance across all domains with large effect sizes for verbal memory, verbal fluency and attention and processing speed. Indeed, cognitive scores fell mostly within 0.5–1.0 SDs below HC data for cognitively impaired participants. Deficits in facial emotion recognition were also significantly greater in cognitively impaired individuals with medium effect sizes, indicating that cluster membership was driven by the degree of impairment across both neurocognitive and social cognitive domains.

Within the CHR-P group, cognitively impaired individuals had significantly poorer functioning than cognitively spared individuals. While role functioning and global functioning were significantly reduced at baseline and follow-up, respectively, social functioning was impaired at both time points, in line with previous findings [29]. Lower levels of premorbid functioning and premorbid IQ were also observed in the cognitively impaired vs. cognitively spared subgroup, consistent with previous studies across the psychosis spectrum [15, 18, 25, 27]. These findings, in addition to the larger number of male participants in our cognitively impaired subgroup, may support the existence of a neurodevelopmental contribution towards pronounced cognitive impairments in CHR-P participants [68], in line with previous results in psychosis patients [69].

In contrast, positive symptom severity did not significantly differ between cognitive subgroups. Cluster analyses have produced mixed findings, reporting either no significant differences across cognitive subgroups in the schizophrenia-spectrum [18, 25, 28, 63] or greater positive symptom severity in the most cognitively impaired cluster [12, 15, 26, 27]. Furthermore, the proportion of CHR-P participants meeting CAARMS criteria, SPI-A criteria or both did not differ between the cognitive subgroups, contrasting with previous reports of lesser cognitive deficits in individuals meeting basic symptom, as opposed to UHR, criteria [70].

### Outcome prediction in the CHR-P group

Importantly, we were also able to predict functional outcome from cluster membership, with cognitively impaired CHR-P individuals significantly more likely to experience poor functional outcome at follow-up. Conversely, cluster membership was unable to predict clinical outcomes in terms of APS persistence or transition to psychosis. This contrasts with Velthorst et al. [29] whereby impaired cognition in CHR-P individuals predicted transition to psychosis. Nevertheless, our findings suggest that early interventions targeting cognition, such as cognitive remediation, should be tailored towards cognitively impaired CHR-P participants to alleviate cognitive deficits and consequently improve functional outcome [71].

### Limitations

Certain limitations should be considered. First, the sample size of FEP participants was small, limiting our ability to accurately characterise cognitive heterogeneity in this group. Furthermore, negative symptoms were not assessed in the current study while cognition was only assessed at baseline. Therefore, we were unable to ascertain the full impact of clinical symptomatology on cluster assignment as well as the stability of cognitive subgroups over time. Finally, cluster membership explained only 8.0% of the variance in functional outcome. This could, in part, be explained by our measure of functioning. For example, the GAF scale confounds functioning with symptom severity, the latter being unrelated to functioning in the current study. Nevertheless, this measure was chosen over social and role functioning scales as these scores were mostly limited in range.

## Conclusions

We employed cluster analysis to investigate cognitive subgroups in CHR-P participants using a community-recruitment approach, social cognitive measures and functional outcome prediction. We identified two discrete cognitive subgroups and found support for considerable cognitive heterogeneity within the CHR-P group. Cognitively impaired and cognitively spared CHR-P individuals could be distinguished on measures of functioning at baseline and follow-up, with cluster membership able to predict functional outcome. These findings emphasise the key role cognition plays in functioning and suggest that cluster assignment is driven by cognitive performance, rather than clinical symptoms. In addition, the current findings may support the role of cognitive enhancement therapies, such as cognitive remediation, in CHR-P individuals with impaired cognition. Indeed, data-driven approaches such as cluster analysis could effectively stratify heterogenous clinical populations along dimensions of interest and thus represent an important step towards personalised psychiatry. Future research should attempt to replicate these findings in larger samples, over longer follow-up periods and also investigate whether these cognitive subgroups are differentially associated with neurobiological measures, such as measures of cortical thickness and volume as well as electrophysiological parameters.

## Supplementary Information

Below is the link to the electronic supplementary material.

Supplementary file1 (PDF 1037 KB)

## Data Availability

Available from the corresponding author on reasonable request.
