# Supplementary material for: Characterising cognitive heterogeneity in individuals at clinical high-risk for psychosis: a cluster analysis with clinical and functional outcome prediction
Source: Eur Arch Psychiatry Clin Neurosci. 2021 Aug 16;272(3):437–48. doi: 10.1007/s00406-021-01315-2 (PMC8938352; doi:10.1007/s00406-021-01315-2)
Supplement: Supplementary file 1 — Supplementary file1 (PDF 1037 KB) [file 406_2021_1315_MOESM1_ESM.pdf]

Characterising cognitive heterogeneity in individuals at clinical high-risk for psychosis: A cluster analysis with clinical and functional outcome prediction

Kate Haining, Ruchika Gajwani , Joachim Gross, Andrew I. Gumley, Robin A. A. Ince, Stephen M. Lawrie, Frauke Schultze-Lutter, Matthias Schwannauer, Peter J. Uhlhaas

European Archives of Psychiatry and Clinical Neuroscience

Corresponding Author:

Dr. Peter J. Uhlhaas

Department of Child and Adolescent Psychiatry

Charité Universitätsmedizin

Berlin, Germany

Email: peter.uhlhaas@charit.de

Tel: 0049 30 450 516 193

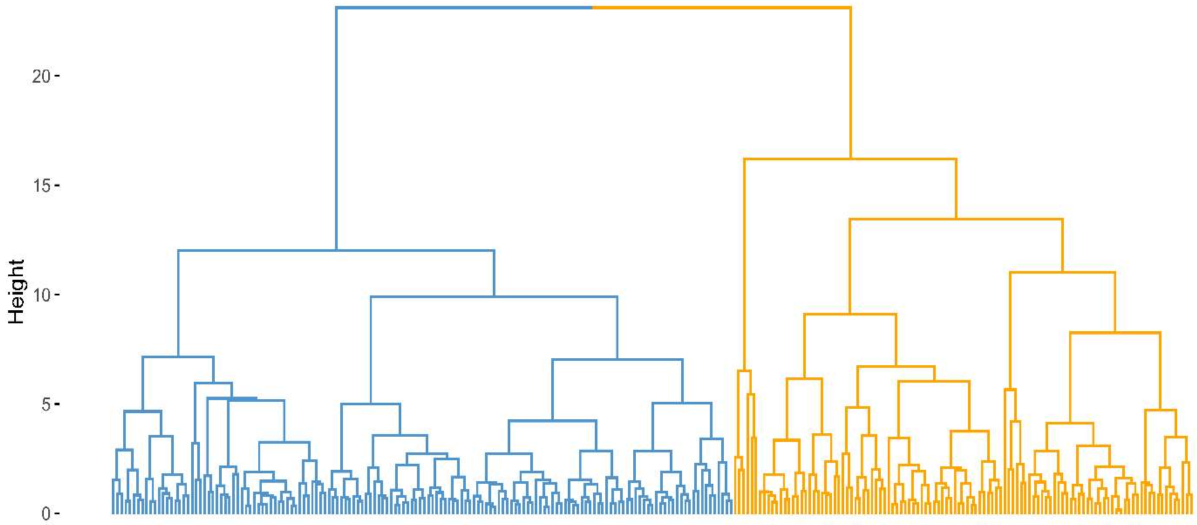

Supplementary Fig. 1. Cluster dendrogram displaying cluster 1 (impaired; orange) and cluster 2 (spared; blue) for the total sample (N = 261). Cluster analysis was conducted using the dist and hclust functions from the *stats* package

Supplementary Table 1. Cognitive domains assessed by the BACS and CNB

BACS cognitive domain	Task	Procedure	Measure	Range
Verbal memory	List learning (version 1)	Participants are read a list of 15 words and then asked to recall as many as possible, in any order. Procedure repeated over five consecutive trials.	Number of words correctly recalled	0-75
Working memory	Digit sequencing task	Participants are read clusters of numbers (e.g. 961) that steadily increase in length. They are asked to recall the numbers in order, from lowest to highest.	Number of correct responses	0-28
Motor speed	Token motor task	Participants are given 100 plastic tokens and asked to place as many as possible in a container, two at a time. Time limit = 60 seconds.	Number of tokens correctly placed in the container	0-100
Verbal fluency	Semantic fluency	Participants are asked to generate as many words as possible within a specific category (i.e. animals). Time limit = 60 seconds.	Number of animals named	0-time variant
	Letter fluency	In two separate trials, participants are asked to produce as many words as possible beginning with a given letter (i.e. F and S). Time limit (per trial) = 60 seconds.	Number of words generated	0-time variant
Attention and processing speed	Symbol coding task	Participants are asked to write the numerals one through nine as matches to non-meaningful symbols on a response sheet as quickly as possible, based on a key provided to them. Time limit = 90 seconds.	Number of correct items	0-110
Executive function	Tower of London (version A)	Participants are shown two pictures (A and B) simultaneously - each showing three balls of different colours uniquely arranged on three pegs. They are asked to estimate the minimum number of times that the balls in picture A would have to be moved in order to match the arrangement in picture B.	Number of correct responses	0-22
**CNB cognitive domain**	**Task**	**Procedure**	**Measure**	**Range**
Attention	Continuous Performance Test-number and letter version (PCPT-nl)	Participants are presented with a series of red vertical and horizontal lines (seven segment displays) that flash in a digital numeric frame (akin to a digital clock). Participants are asked to press the spacebar when the lines form a complete number (initial 3 mins) or a complete letter (next 3 mins). Each stimulus is shown for 300 ms followed by a blank screen for 700 ms, allowing the participant 1 s to respond per trial.	Number of true positive responses	0-120
			Median response time for true positive responses	0-1000ms
Working memory	Letter-N-Back (LNB2)	Participants are presented with a continual series of flashing letters, one at a time, and asked to press the spacebar according to three different rules. In the 0-back condition, the spacebar must be pressed whenever the letter “X” appears. In the 1-back condition, the spacebar must be pressed whenever the current letter matches the previous letter. In the 2-back condition, the spacebar must be pressed	Number of correct responses	0-45
			Median response time for correct responses	0-2500ms

		whenever the current letter matches the letter before the previous letter. Each stimulus is shown for 500 ms followed by a blank screen for 2000 ms, allowing the participant 2.5 s to respond per trial.		
Emotion recognition	Emotion Recognition Task (ER40)	Participants are presented with 40 colour photographs of faces, one at a time, and asked to determine the specific emotion being expressed from five possible choices: happy, sad, anger, fear or no emotion. Participants respond by clicking on their chosen emotion with the mouse.	Number of correct responses	0-40
			Median response time for correct responses	0-time variant

Note: BACS, Brief Assessment of Cognition in Schizophrenia; CNB, Penn Computerized Neurocognitive Battery

Supplementary Table 2. Pattern matrix for the total sample (N = 261)

Measure	Oblimin rotated component loadings				
	VF	ER	ATT	WM	GCF
Verbal memory	0.19	-0.05	0.08	0.07	**0.61**
WM	0.37	-0.16	-0.11	0.12	**0.59**
Motor speed	-0.24	0.37	0.28	-0.07	0.36
VF semantic	**0.75**	0.07	-0.02	0.07	0.01
VF letter F	**0.85**	-0.01	0.04	-0.11	0.02
VF letter S	**0.82**	0.03	0.08	0.02	-0.04
VF total	**0.96**	0.02	0.02	0.02	0.02
ATT & processing speed	0.08	0.29	0.12	-0.01	**0.63**
Executive function	-0.19	-0.03	0.04	0.09	**0.70**
ER efficiency	0.01	**0.89**	0.00	0.10	0.03
ER anger efficiency	0.06	**0.74**	-0.06	-0.13	0.09
ER happy efficiency	0.07	**0.72**	0.15	0.02	-0.20
ER fear efficiency	0.00	**0.50**	-0.11	0.37	-0.12
ER sad efficiency	0.05	**0.74**	-0.07	0.03	0.07
WM efficiency	0.00	0.01	0.08	**0.93**	0.02
WM 1-back efficiency	-0.02	-0.02	-0.03	**0.89**	-0.02
WM 2-back efficiency	-0.01	0.05	0.05	**0.80**	0.06
ATT efficiency	0.02	-0.02	**0.97**	0.02	0.01
ATT letter efficiency	0.03	0.00	**0.91**	0.01	-0.06
ATT number efficiency	0.02	-0.01	**0.86**	0.05	0.08
Eigenvalues	3.26	3.04	2.79	2.65	1.96
% of variance	16	15	14	13	10
α	.89	.82	.93	.88	.68

Note: Component loadings over .40 appear in bold. VF, verbal fluency; ER, emotion recognition; ATT, attention; WM, working memory; GCF, general cognitive function

Supplementary Table 3. Structure matrix for the total sample (N = 261)

Measure	Oblimin rotated component loadings				
	VF	ER	ATT	WM	GCF
Verbal memory	0.36	0.08	0.31	0.27	**0.69**
WM	**0.47**	-0.02	0.16	0.23	**0.67**
Motor speed	-0.02	0.40	0.35	0.22	0.39
VF semantic	**0.77**	0.22	0.25	0.19	0.21
VF letter F	**0.85**	0.11	0.25	0.03	0.20
VF letter S	**0.85**	0.19	0.33	0.16	0.19
VF total	**0.98**	0.20	0.33	0.17	0.27
ATT & processing speed	0.31	0.38	0.35	0.30	**0.70**
Executive function	-0.01	0.04	0.18	0.25	**0.68**
ER efficiency	0.18	**0.93**	0.25	**0.41**	0.14
ER anger efficiency	0.17	**0.70**	0.09	0.13	0.13
ER happy efficiency	0.19	**0.76**	0.29	0.28	-0.07
ER fear efficiency	0.07	**0.59**	0.12	**0.47**	-0.01
ER sad efficiency	0.17	**0.75**	0.14	0.28	0.14
WM efficiency	0.16	0.35	**0.45**	**0.97**	0.28
WM 1-back efficiency	0.09	0.27	0.30	**0.86**	0.20
WM 2-back efficiency	0.15	0.34	0.38	**0.85**	0.28
ATT efficiency	0.31	0.21	**0.98**	0.39	0.25
ATT letter efficiency	0.29	0.20	**0.91**	0.35	0.17
ATT number efficiency	0.30	0.21	**0.90**	0.40	0.30

Note: Component loadings over .40 appear in bold. VF, verbal fluency; ER, emotion recognition; ATT, attention; WM, working memory; GCF, general cognitive function

Supplementary Table 4. Cognitive characteristics of the CHR-P group by cognitive cluster at baseline (N = 146)

Measure	Cluster 1		Cluster 2		*p*	Effect size[a]
	Impaired (N= 67)		Spared (N = 79)			
	Mean	SD	Mean	SD		
Premorbid IQ[b]	108.23	7.64	111.39	6.00	.007	d = 0.460
Verbal memory	-0.72	1.10	0.24	1.11	< .001	d = 0.873
WM	-0.60	1.31	0.41	1.31	< .001	r = 0.377
Motor speed	-1.13	1.22	-0.30	1.07	< .001	d = 0.729
VF semantic	-0.63	0.93	0.44	1.04	< .001	d = 1.087
VF letter F	-0.67	0.93	0.28	1.01	< .001	r = 0.458
VF letter S	-0.78	1.08	0.37	1.29	< .001	r = 0.446
VF total	-0.80	0.91	0.56	1.11	< .001	r = 0.575
ATT & processing speed	-1.12	0.82	0.14	1.09	< .001	r = 0.581
Executive function	-0.44	1.45	0.33	1.14	< .001	r = 0.290
ER efficiency	-0.87	1.15	0.06	0.62	< .001	r = 0.472
ER anger efficiency	-0.42	1.04	0.20	0.57	< .001	r = 0.360
ER happy efficiency	-0.83	1.09	-0.10	0.76	< .001	r = 0.391
ER fear efficiency	-0.36	1.33	0.13	0.59	< .001	r = 0.337
ER sad efficiency	-0.58	1.08	0.15	0.64	< .001	r = 0.393
WM efficiency	-0.47	1.10	0.13	0.63	< .001	r = 0.314
WM 1-back efficiency	-0.37	1.24	0.14	0.72	.005	r = 0.231
WM 2-back efficiency	-0.43	0.89	0.07	0.60	< .001	r = 0.302
ATT efficiency	-0.78	1.40	0.20	0.68	< .001	r = 0.443
ATT letter efficiency	-0.50	1.22	0.29	0.78	< .001	r = 0.394
ATT number efficiency	-0.73	1.45	0.25	0.60	< .001	r = 0.396

Note: VF, verbal fluency; ER, emotion recognition; ATT, attention; WM, working memory; GCF, general cognitive function

[a] Effect sizes were Rosenthal's r for Mann-Whitney U tests (small effect = 0.1, medium effect = 0.3, large effect = 0.5) and Cohen's d for Welch's t-tests (small effect = 0.2, medium effect = 0.5, large effect = 0.8)

[b] This measure was not included in the PCA or cluster analysis

Supplementary Results

Following exclusion of the FEP group, the aforementioned principal components were re-extracted and, in combination, explained 67% of the variance in cognitive performance (Supplementary Fig. 2). As before, agglomerative hierarchical clustering with 2 clusters was favoured, resulting in the emergence of a cognitively impaired (n = 105; 42.7%) and spared (n = 141; 57.3%) cluster (Supplementary Fig. 3). Linear discriminant analysis confirmed that we were able to predict the cluster membership of new cases with a mean accuracy of 87.5%. Cluster 1 comprised 50.0% of CHR-P participants, 31.9% of CHR-N participants and 32.1% of HCs (Supplementary Fig. 4).

Group differences remained relatively unchanged, with CHR-P individuals in cluster 1 displaying significantly poorer performance across all 20 cognitive tests ($p < .05$) as well as impairments in social ($p = .036$; r = 0.174), role ($p = .029$; r = 0.180) and premorbid ($p = < .001$, r = 0.337) functioning (Supplementary Fig. 5). Male CHR-P participants were still significantly more likely ($p < .001$; $\phi = 0.303$) to be allocated to cluster 1 (42.5%) than cluster 2 (15.1%). Similar impairments were also evident at follow-up with poor functional outcome significantly more likely ($p = .006$, $\phi = 0.247$) in cluster 1 (71.0%) than cluster 2 (46.7%) and CHR-P individuals in cluster 1 displaying poorer global ($p = .020$; r = .210) and social ($p = .045$; r = .182) functioning. Finally, cluster membership explained 8.1% of the variance in functional outcome ($p = .007$, AUC = 0.626, sensitivity = 61.1% and specificity = 64.0) but was unable to predict CAARMS persistence ($p = .788$) or transition to psychosis ($p = .290$).

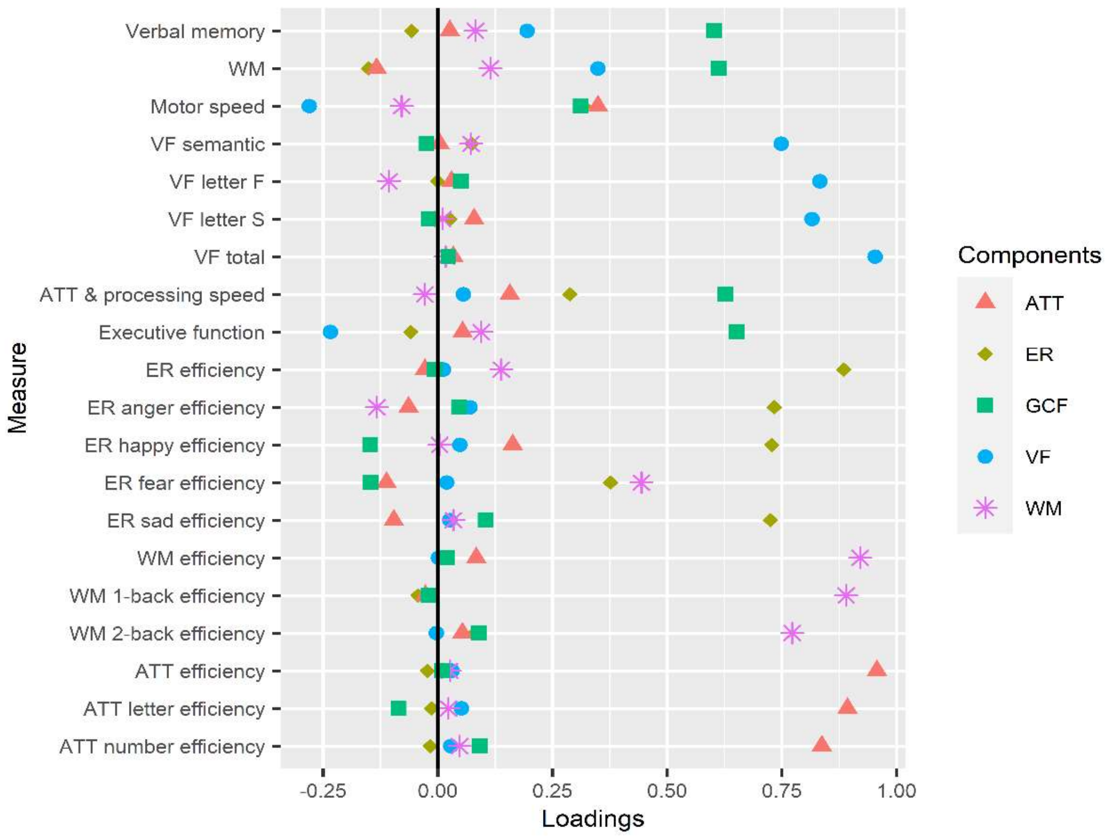

Supplementary Fig. 2. Component loading plot following exclusion of the FEP group (N = 246). ATT, attention; ER, emotion recognition; GCF, general cognitive function; VF, verbal fluency; WM, working memory

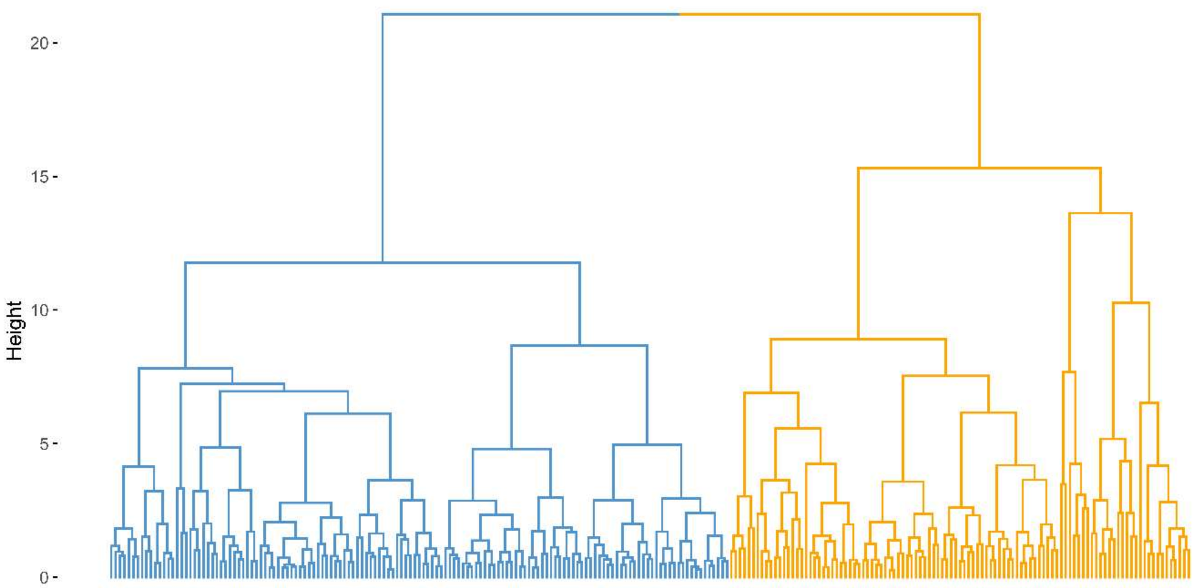

Supplementary Fig. 3. Cluster dendrogram displaying cluster 1 (impaired; orange) and cluster 2 (spared; blue) following exclusion of the FEP group (N = 246). Cluster analysis was conducted using the dist and hclust functions from the *stats* package

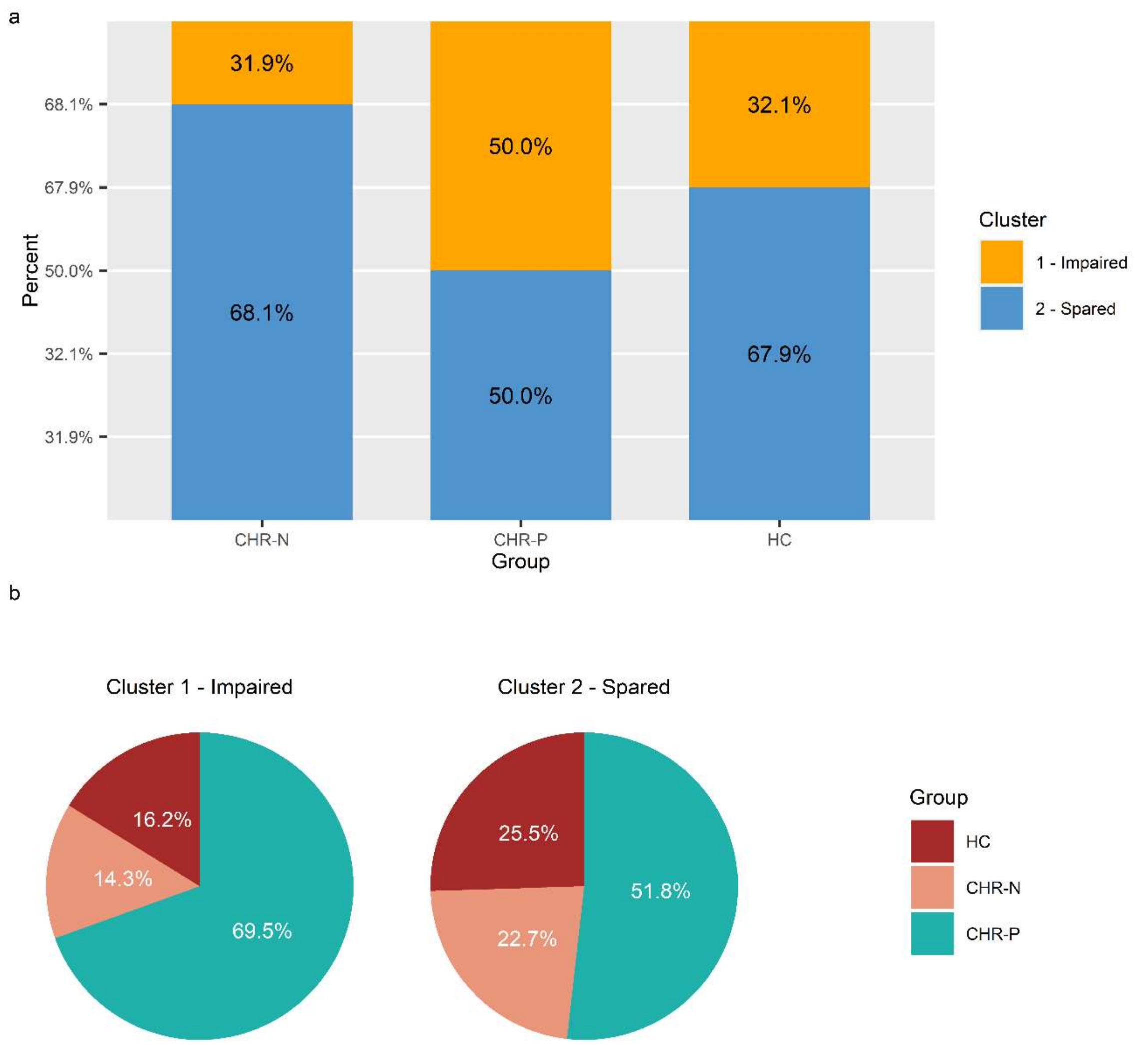

Supplementary Fig. 4. The distribution of (a) clusters within each diagnostic group and (b) diagnostic groups within each cluster following exclusion of the FEP group (N = 246). CHR-P, clinical high-risk for psychosis; CHR-N, clinical high-risk-negative; HC, healthy control

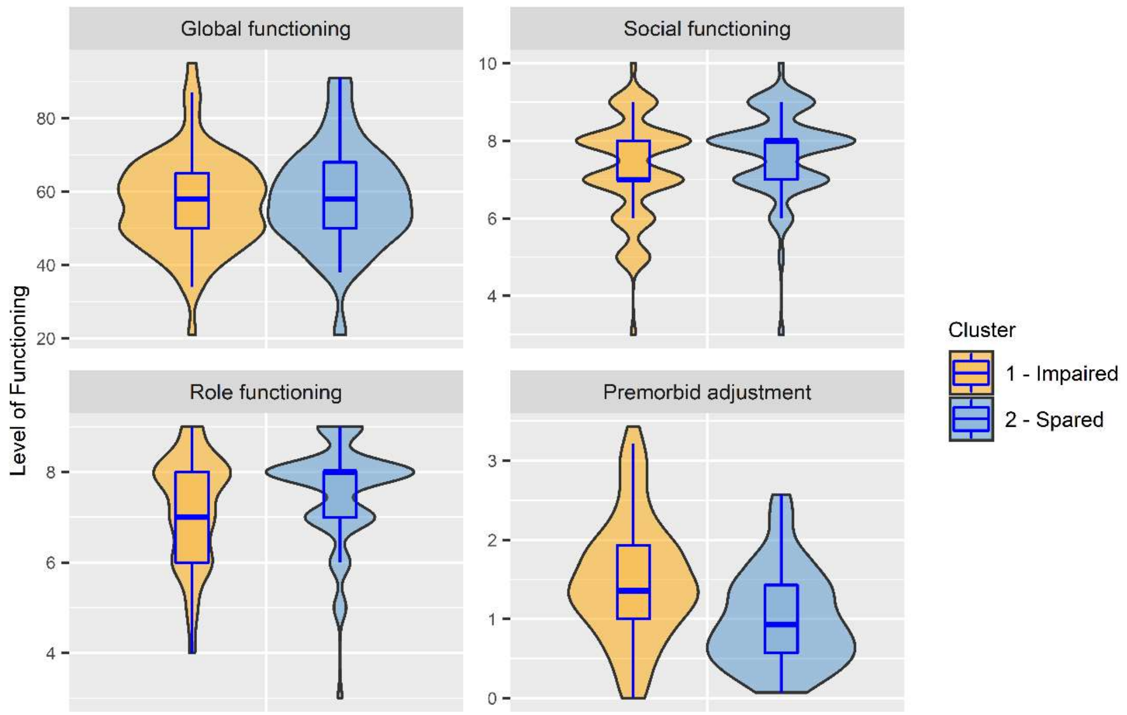

Supplementary Fig. 5. Level of functioning across cognitive clusters for the CHR-P group (N = 146), formed following exclusion of the FEP group. CHR-P individuals in cluster 1 were characterised by poorer social, role and premorbid functioning ($p < .05$) but not global functioning ($p = .528$)
